# Sex-Specific Misclassification of Obesity When Using Body Mass Index in Young Healthcare Professionals: A Large Cross-Sectional Study Using Multiple Adiposity Indices

**DOI:** 10.3390/medsci14020234

**Published:** 2026-05-01

**Authors:** Alberto Ramirez Gallegos, Pedro Juan Tárraga López, Mónica Silu Piña Dabreu, Lluis Rodas Cañellas, Ángel Arturo López-González, José Ignacio Ramírez-Manent

**Affiliations:** 1 Balearic Islands Health Service, 07010 Palma, Spain; alberto.ramirez@ibsalut.es (A.R.G.); joseignacio.ramirez@ibsalut.es (J.I.R.-M.); 2 Faculty of Medicine, University of Castilla La Mancha (UCLM), 02008 Albacete, Spain; pjtarraga@sescam.jccm.es; 3 ADEMA-University School, University of the Balearic Islands, 07009 Palma, Spain; m.pina@eua.edu.es (M.S.P.D.); ll.rodas@eua.edu.es (L.R.C.); 4 Faculty of Medicine, University of the Balearic Islands, 07120 Palma, Spain

**Keywords:** obesity misclassification, visceral adiposity, body mass index, healthcare professionals, sex differences, Mediterranean diet, waist-to-height ratio

## Abstract

**Background**: Body mass index (BMI) remains the standard tool for obesity screening; however, it does not account for body fat distribution or visceral adiposity, potentially leading to clinically relevant misclassification—particularly in young adults. Evidence on this issue in healthcare professionals is limited. **Objective**: To evaluate the extent of obesity misclassification when using BMI compared with alternative anthropometric and body composition indices, and to examine sex-specific associations between lifestyle factors and different adiposity phenotypes in young healthcare professionals. **Methods**: A large cross-sectional study was conducted in 12,874 medical residents, nursing residents, and age-matched controls (22–30 years). Obesity was defined using BMI (≥30 kg/m^2^), waist-to-height ratio (WtHR ≥ 0.5), Clínica Universidad de Navarra–Body Adiposity Estimator (CUN-BAE), body fat percentage, and bioimpedance-derived visceral fat. Multivariable logistic regression models adjusted for age, sex, professional group, smoking, physical activity, and Mediterranean diet adherence were fitted separately for each adiposity definition. Sex interaction terms were formally tested. Agreement between indices was assessed using Cohen’s kappa. **Results**: Obesity prevalence varied substantially according to the index applied and was consistently higher when central or visceral adiposity measures were used. Agreement between BMI and alternative indices was only fair to moderate, with the lowest concordance observed for visceral fat (κ = 0.29; 95% CI 0.26–0.32). Male sex was strongly associated with visceral fat-defined obesity (aOR 4.76; 95% CI 3.82–5.92), while effect sizes were attenuated for BMI-defined obesity (aOR 1.41; 95% CI 1.32–1.51). Significant sex interactions were detected for visceral adiposity, particularly for physical activity (*p* = 0.001) and smoking (*p* = 0.002), indicating differential lifestyle associations according to fat distribution phenotype. **Conclusions**: BMI substantially underestimates clinically relevant central and visceral adiposity in young healthcare professionals. Sex-specific differences were observed in the association between lifestyle behaviors and visceral fat. These findings highlight the limitations of relying exclusively on BMI for obesity screening. Incorporating waist-based or body composition-derived measures may improve early risk identification and support targeted preventive strategies.

## 1. Introduction

Obesity has emerged as one of the most significant global public health challenges of the 21st century. According to the World Health Organization, its prevalence has nearly tripled since 1975, currently affecting more than 650 million adults worldwide [1]. Beyond its well-established association with cardiovascular disease, type 2 diabetes, and certain malignancies [2,3], obesity contributes substantially to premature mortality, functional impairment, and increased healthcare utilization. Importantly, recent epidemiological evidence suggests that the burden of obesity-related morbidity is increasingly observed in younger populations, raising concerns about early cardiometabolic risk accumulation [4,5].

Traditionally, obesity has been defined using body mass index (BMI), a simple and widely adopted anthropometric measure. Although BMI remains practical for population-level surveillance, its limitations as a surrogate of adiposity are increasingly recognized [6,7]. BMI does not differentiate between fat mass and lean mass, nor does it account for fat distribution—an aspect that plays a crucial role in cardiometabolic risk stratification [8,9,10]. Consequently, individuals with elevated body fat or central adiposity may be misclassified as non-obese. This occurs when BMI is used as the sole diagnostic criterion.

Growing evidence indicates that central and visceral adiposity are more strongly associated with insulin resistance, hypertension, dyslipidemia, and cardiovascular events than overall adiposity estimated by BMI alone [8,9,10]. Abdominal fat depots exhibit greater metabolic activity. This includes increased lipolytic flux and pro-inflammatory cytokine secretion. These mechanisms contribute to adverse metabolic profiles [8,9]. These pathophysiological mechanisms support the proposal that indices reflecting central fat distribution may offer superior cardiometabolic risk discrimination [11,12,13].

Among alternative anthropometric indicators, the waist-to-height ratio (WtHR) has been suggested as a simple and robust screening tool across age groups and ethnicities [11]. Similarly, body composition-based estimators such as the Clínica Universidad de Navarra–Body Adiposity Estimator (CUN-BAE) incorporate age and sex to improve adiposity estimation beyond BMI [14,15]. The ongoing debate regarding the adequacy of BMI as the primary diagnostic tool for obesity reflects the need to reassess screening strategies, particularly in populations where fat distribution patterns may differ substantially [16].

Lifestyle factors remain central determinants of obesity development and progression. Physical inactivity, smoking, and suboptimal dietary patterns are consistently associated with increased adiposity and adverse metabolic outcomes [17,18,19]. Conversely, adherence to dietary patterns such as the Mediterranean diet has been linked to lower body weight and improved cardiometabolic profiles [20,21]. However, the strength and direction of these associations may vary depending on how adiposity is defined, as different indices capture distinct physiological aspects of fat accumulation.

Healthcare professionals represent a particularly relevant population in this context. Despite their medical knowledge and preventive role, physicians and nurses are not immune to overweight and obesity [22,23]. Medical and nursing residency programs are characterized by long working hours, high stress exposure, and sleep restriction, all of which have been associated with weight gain and metabolic disturbances [24,25]. Moreover, the health behaviors of healthcare professionals may influence their patient counseling practices and credibility in promoting healthy lifestyles [26].

Importantly, biological sex influences fat distribution and metabolic risk. Men generally accumulate more visceral adiposity, whereas women preferentially store subcutaneous fat [27,28]. These differences may modify the association between lifestyle behaviors and adiposity phenotypes, yet many epidemiological studies rely solely on BMI without formally evaluating sex-specific heterogeneity.

Despite increasing recognition of BMI limitations, few studies have comprehensively evaluated obesity using multiple anthropometric and body composition indices in young healthcare professionals. Even fewer have examined whether associations between lifestyle factors and obesity differ according to adiposity definition or whether sex acts as an effect modifier in these relationships.

In this context, the present study aims to assess obesity using multiple anthropometric and adiposity indices and to examine their association with health behaviors among medical and nursing residents. By comparing traditional BMI-based classification with alternative indices reflecting central and visceral adiposity, this study seeks to clarify whether reliance on BMI alone may underestimate clinically relevant adiposity in young healthcare professionals and to explore potential sex-specific differences in these associations.

## 2. Methods

### 2.1. Study Design and Population

A cross-sectional study was conducted among medical and nursing residents enrolled in accredited residency programs, together with an age-matched control group of non-healthcare young adults. Participants were consecutively recruited through institutional occupational health services during routine mandatory health assessments.

Eligible individuals were between 22 and 30 years of age and were either actively enrolled in a residency training program or employed in a comparable professional setting (control group). Exclusion criteria included previously diagnosed endocrine disorders affecting body composition (e.g., Cushing syndrome or untreated thyroid disease), pregnancy, or chronic systemic disease requiring long-term corticosteroid therapy.

All eligible individuals attending occupational health examinations during the study period were invited to participate, and no sampling was performed. The final sample therefore reflects the underlying working population assessed during the recruitment window.

The study was conducted in accordance with the principles of the Declaration of Helsinki [29]. The protocol was approved by the Institutional Research Ethics Committee, and written informed consent was obtained from all participants prior to inclusion.

Study design and reporting adhered to the Strengthening the Reporting of Observational Studies in Epidemiology (STROBE) guidelines for cross-sectional studies [30].

### 2.2. Anthropometric and Clinical Assessment

All anthropometric measurements were performed by trained healthcare personnel following standardized procedures to minimize inter-observer variability [31].

Body weight was measured to the nearest 0.1 kg using a calibrated digital scale SECA 700 (SECA, Chino, CA, USA), with participants wearing light clothing and no shoes. Height was measured to the nearest 0.1 cm using a wall-mounted stadiometer SECA 220 (SECA, Chino, CA, USA). Body mass index (BMI) was calculated as weight (kg) divided by height squared (m^2^) and classified according to World Health Organization criteria [32].

Waist circumference was measured midway between the lowest rib and the iliac crest at the end of normal expiration. Hip circumference was measured at the level of the greater trochanters with a SECA tape measure (SECA, Chino, CA, USA). Waist-to-height ratio (WtHR) and waist-to-hip ratio were calculated accordingly. Cut-off values for central obesity were defined according to internationally accepted thresholds [33].

Body composition parameters, including total body fat percentage and visceral fat percentage, were obtained using bioelectrical impedance analysis (BIA). Measurements were performed under standardized conditions (overnight fasting, bladder emptied, and no vigorous physical activity within 12 h), in accordance with manufacturer recommendations [34]. Although dual-energy X-ray absorptiometry (DXA) is considered a reference standard, BIA has demonstrated acceptable validity for population-based studies when standardized protocols are applied [35].

The Clínica Universidad de Navarra–Body Adiposity Estimator (CUN-BAE) was calculated using the validated equation incorporating age, sex, and BMI [36].

Blood pressure was measured in the seated position after five minutes of rest using an automated sphygmomanometer OMRON-M3 monitor (OMRON, Osaka, Japan). Venous blood samples were obtained after overnight fasting for biochemical assessment, including glucose and lipid profile, using standardized laboratory methods.

### 2.3. Lifestyle Variables

Smoking status was self-reported and categorized as current smoker (yes/no).

Physical activity was assessed using the short form of the International Physical Activity Questionnaire (IPAQ), a validated instrument for population-based studies [37]. Total weekly physical activity was calculated according to IPAQ scoring protocols and expressed in minutes per week. Participants were classified as physically active if they met current international recommendations (≥150 min/week of moderate-intensity activity, ≥75 min/week of vigorous-intensity activity, or an equivalent combination), in accordance with global physical activity guidelines [38].

Adherence to the Mediterranean diet was evaluated using the 14-item Mediterranean Diet Adherence Screener (MEDAS), originally developed and validated within the PREDIMED framework [39]. Each item was scored as 0 or 1, yielding a total score ranging from 0 to 14. Participants were classified as adherent when the score was ≥9 points, consistent with prior epidemiological research [40].

### 2.4. Definition of Obesity According to Different Indices

Obesity was defined separately according to established and widely used cut-off values for each index. Specifically, BMI-defined obesity was considered as ≥30 kg/m^2^. Central obesity was defined using a waist-to-height ratio (WtHR) ≥ 0.5. CUN-BAE-defined obesity was classified according to validated sex-specific thresholds as described in the original validation studies. High body fat percentage was defined using sex-specific cut-offs (≥25% in men and ≥35% in women), and elevated visceral fat was defined according to manufacturer-recommended and published reference values based on bioelectrical impedance analysis [41].

Each adiposity index was analyzed independently as a binary outcome variable. Indices were not included simultaneously in the same multivariable model to avoid collinearity due to shared anthropometric components.

### 2.5. Statistical Analysis

Statistical analyses were performed using SPSS version 30.0 (IBM Corp., Armonk, NY, USA).

Continuous variables are presented as mean and standard deviation (SD), and categorical variables as frequencies and percentages. Normality assumptions were assessed graphically and using the Kolmogorov–Smirnov test. For the analyses presented in Table 1 and Table 2, continuous variables were evaluated using two-way analysis of variance (ANOVA), with sex and professional group included as fixed factors, together with the sex × group interaction term. Categorical variables were analyzed using log-linear models including sex, professional group, and their interaction. When significant group effects were identified, post hoc pairwise comparisons between professional groups were performed using Bonferroni correction.

Multivariable logistic regression models were constructed to evaluate the independent association between lifestyle behaviors and obesity defined according to each adiposity index. Separate models were fitted for each outcome (BMI-defined obesity, high WtHR, CUN-BAE obesity, very high body fat percentage, high visceral fat, and clinical obesity).

All models were adjusted for age (continuous), sex, professional group (medical residents, nursing residents, control group), smoking status (yes/no), physical activity (yes/no), and Mediterranean diet adherence (yes/no). Adjusted odds ratios (aORs) and 95% confidence intervals (95% CIs) were reported. A composite definition of clinical obesity was created based on the presence of abnormal values in central or body composition-derived adiposity indices.

To explore sex-specific associations, regression analyses were repeated separately for men and women. Effect modification by sex was formally evaluated by including multiplicative interaction terms (sex × smoking, sex × physical activity, and sex × Mediterranean diet adherence) in the fully adjusted models. Statistical significance of interaction terms was assessed using the Wald test.

Agreement between obesity classifications derived from different adiposity indices was assessed using Cohen’s kappa coefficient (κ) for categorical variables. Kappa values were interpreted according to Landis and Koch criteria: <0.20 poor, 0.21–0.40 fair, 0.41–0.60 moderate, 0.61–0.80 substantial, and >0.80 almost perfect agreement.

All statistical tests were two-sided, and a *p*-value < 0.05 was considered statistically significant.

## 3. Results

### 3.1. Baseline Characteristics

A total of 12,874 participants were included in the analysis. Baseline characteristics stratified by sex and professional group are presented in Table 1. Continuous variables were analyzed using two-way ANOVA, including sex, professional group, and the sex × group interaction term. Categorical variables were assessed using log-linear models with the same factors. Corresponding *p*-values for sex, group, and interaction are shown in Table 1. When appropriate, post hoc pairwise comparisons with Bonferroni correction were performed to identify specific group differences.

Overall, significant main effects for sex and professional group were observed across most variables. Healthcare residents generally exhibited more favorable cardiometabolic profiles compared with the control group. In addition, several variables showed significant sex × group interactions, indicating that differences between professional groups were not consistent between men and women.

Most variables showed significant main effects for sex and professional group. In addition, several variables showed significant sex × group interactions, indicating that differences between professional groups were not uniform across men and women.

### 3.2. Distribution of Adiposity Indices

Anthropometric and body composition indices are summarized in Table 2. Continuous variables were analyzed using two-way ANOVA, including sex, professional group, and the sex × group interaction term. Corresponding *p*-values for sex, group, and interaction are shown in Table 2. Most indices showed significant main effects for sex and professional group. In addition, several indices showed significant sex × group interactions, indicating that differences between professional groups varied according to sex.

### 3.3. Variability in Obesity Classification

The prevalence of obesity according to different definitions is presented in Table 3 and was analyzed using log-linear models including sex, professional group, and obesity definition, together with their interaction terms.

BMI-defined obesity showed the lowest prevalence across most groups, whereas alternative measures such as CUN-BAE and body fat percentage identified a greater proportion of individuals as obese. Visceral fat-defined obesity, although less frequent overall, demonstrated marked sex differences.

These findings indicate substantial variability in obesity classification depending on the adiposity index used.

### 3.4. Multivariable Associations with Adiposity Indices

**Figure 1:**
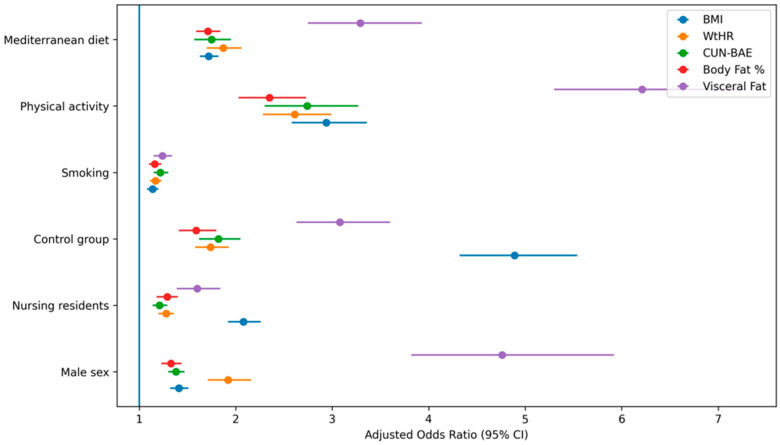
Combined forest plot of multivariable-adjusted associations between lifestyle factors, professional group, and obesity across adiposity indices. Adjusted odds ratios (aORs) and 95% confidence intervals (95% CIs) for obesity defined by body mass index (BMI), waist-to-height ratio (WtHR), CUN-BAE, body fat percentage, and visceral fat. All models were adjusted for age, sex, professional group, smoking status, physical activity, and Mediterranean diet adherence. Reference categories were female sex, medical residents, non-smokers, physically active participants, and adherence to the Mediterranean diet.

Multivariable-adjusted associations between lifestyle factors and obesity defined according to each adiposity index are presented in Table 4 and Figure 1.

Male sex was independently associated with higher odds of obesity across all indices, with the strongest association observed for visceral fat-defined obesity (aOR 4.76; 95% CI 3.82–5.92). In contrast, the magnitude of association was attenuated when obesity was defined using BMI (aOR 1.41; 95% CI 1.32–1.51).

Belonging to the control group was consistently associated with increased odds of obesity across all definitions, particularly for visceral fat (aOR 3.08; 95% CI 2.63–3.60).

Lifestyle behaviors were also independently associated with adiposity. Physical inactivity and non-adherence to the Mediterranean diet were strongly associated with all obesity phenotypes, with effect sizes again more pronounced for visceral fat.

In addition, a model based on a composite definition of clinical obesity was included, showing consistent associations with the studied factors and slightly stronger effect sizes compared with BMI-defined obesity.

Figure 1 presents the multivariable-adjusted associations between sex, professional group, and lifestyle factors with obesity across the different adiposity indices evaluated. Overall, the direction of associations was consistent across indices; however, effect sizes varied substantially depending on the adiposity definition used.

Visceral fat-defined obesity showed the strongest associations, particularly for male sex and physical activity. This indicates a differential relationship between lifestyle factors and central adiposity compared with BMI. In contrast, BMI-derived estimates demonstrated comparatively attenuated effect sizes, suggesting potential underestimation of adiposity-related risk when relying solely on traditional anthropometric definitions.

### 3.5. Agreement Between Adiposity Indices

Agreement with the clinical obesity definition was moderate to substantial across most adiposity indices, with the highest concordance observed for body fat percentage. (Table 5).

The lowest concordance was observed between BMI and visceral fat-defined obesity (κ = 0.29; 95% CI 0.26–0.32), whereas indices incorporating body composition showed higher levels of agreement. These findings suggest that BMI may inadequately capture central and visceral adiposity in this population.

### 3.6. Sex-Stratified Analyses and Interaction Testing

Sex-stratified models (Table 6) revealed that associations between lifestyle factors and obesity were generally stronger in men, particularly for visceral adiposity. For example, physical inactivity was associated with markedly higher odds of visceral fat-defined obesity in men compared with women.

These patterns were consistent when applying the clinical obesity definition, with slightly stronger associations observed for physical inactivity and professional group in both sexes.

Formal interaction testing confirmed significant sex × lifestyle interactions for visceral adiposity (Table 7), particularly for physical activity (*p* = 0.001) and smoking (*p* = 0.002). These interaction analyses formally test differences in odds ratios between strata (men vs. women), supporting the observed heterogeneity in the sex-stratified models.

These findings indicate that sex-related heterogeneity is more pronounced when central or visceral adiposity is considered rather than BMI alone.

To formally assess whether associations differed by sex, interaction terms between sex and lifestyle factors were included in the fully adjusted models (Table 7).

Significant interaction by sex was observed primarily for visceral fat-defined obesity, particularly for physical activity and smoking. These findings indicate that the relationship between lifestyle factors and central adiposity differs between men and women. In contrast, interaction terms were not statistically significant for BMI-defined obesity, suggesting that sex-related heterogeneity is more pronounced when central or visceral adiposity is considered.

## 4. Discussion

### 4.1. Principal Findings

This large cross-sectional study in young healthcare professionals shows that obesity prevalence varies substantially depending on the adiposity index applied, with BMI yielding consistently lower estimates than indices incorporating body fat or visceral adiposity. The fair agreement between BMI and visceral fat-defined obesity suggests that BMI may miss clinically relevant adiposity phenotypes. In multivariable models, lifestyle factors were consistently associated with obesity across definitions, but effect sizes were markedly stronger for visceral adiposity, highlighting phenotype-specific behavioral correlates.

Additionally, the inclusion of a composite definition of clinical obesity allowed a more comprehensive assessment of adiposity-related risk beyond traditional BMI-based classification.

In addition, the differences observed between medical residents, nursing residents, and the control group deserve further discussion. In the present study, healthcare residents showed more favorable cardiometabolic profiles compared with the control group, despite the similar age range. This finding may be explained by differences in health-related behaviors and awareness. Healthcare professionals are likely to have greater knowledge of preventive strategies and may be more engaged in healthier lifestyles. These associations remained consistent when using the clinical obesity definition, with slightly stronger effect sizes observed particularly for physical inactivity and professional group. In contrast, the control group may not have the same level of health awareness or structured health monitoring. These factors could contribute to the differences observed between groups.

### 4.2. Comparison with Previous Research and Obesity Misclassification

Our findings align with evidence indicating that BMI-based categorization has limited accuracy for identifying obesity when compared with body fat-based definitions and can lead to substantial misclassification [42,43]. This limitation has been further supported by work emphasizing that commonly used adiposity proxies (BMI, percent body fat, and related markers) capture partially different constructs [44] and by recent critical discussions proposing more comprehensive diagnostic approaches beyond BMI alone [45,46]. Additional population-based data also support that BMI may have imperfect diagnostic accuracy for obesity in clinical settings [47] and that combining BMI with central obesity measures improves cardiovascular risk stratification [48,49].

### 4.3. Why Waist-Based and Visceral Measures Show Stronger Associations

The stronger associations observed for central and visceral adiposity measures are consistent with consensus-driven recommendations that waist circumference should be treated as a clinically meaningful “vital sign” [50] and with earlier consensus statements linking waist circumference more directly to cardiometabolic risk [51]. Contemporary work continues to highlight the complexity of interpreting waist measures and their mechanistic relevance to hypertension risk [52]. In addition, evidence from large cohorts shows that visceral and intrahepatic fat are strongly associated with cardiometabolic risk beyond other ectopic depots [53,54], and emerging syntheses extend the relevance of adipose depots to vascular remodeling pathways [55].

### 4.4. Plausibility of the High OR for Physical Inactivity and Visceral Adiposity

The notably high odds ratio observed for physical inactivity in relation to visceral fat-defined obesity is biologically plausible. Differences in adipose tissue function and metabolic activity help explain why sedentary behavior may be more tightly linked to visceral fat than to BMI-defined obesity [56]. Experimental and mechanistic work also supports that adiposity and metabolic regulation can show depot-specific responses under different biological contexts [57]. At the population level, sedentary behavior has been associated with abdominal obesity and downstream healthcare costs, reinforcing the relevance of inactivity specifically to central adiposity phenotypes [58,59]. Taken together, these data support the interpretation that the large effect size reflects a phenotype-specific link between inactivity and visceral adiposity rather than being solely a statistical artifact.

### 4.5. Smoking, Diet, and the Occupational Context

Associations observed for smoking are consistent with evidence that tobacco exposure influences cardiometabolic risk profiles and risk-scale values in large working populations [60]. The inverse association between Mediterranean diet adherence and obesity phenotypes aligns with interventional and observational evidence showing beneficial effects of Mediterranean-style dietary patterns on body weight and cardiometabolic risk factors [61] and with findings connecting Mediterranean diet adherence to metabolic liver disease pathways in at-risk populations [62].

Although healthcare workers are often assumed to have healthier behaviors, residency and early-career stages may still be characterized by unfavorable weight trajectories. Prior work has documented overweight among physicians during residency [63], and more recent evidence links poor sleep quality with overweight/obesity in healthcare professionals [64], which may contribute to central fat accumulation patterns in this occupational group.

### 4.6. Clinical Implications: Normal-Weight Obesity and Sex-Related Heterogeneity

The gap between BMI-defined obesity and adiposity-based definitions is clinically relevant because individuals may present a “normal-weight obesity” phenotype—normal BMI but elevated fat mass and cardiometabolic risk—supported by systematic reviews and meta-analytic evidence [65] and by studies highlighting the role of atherogenic lipoprotein profiles and insulin sensitivity in risk prediction within this phenotype [66]. Sex-related biological differences in adipose tissue biology and energy metabolism may further shape fat distribution and risk expression [67], potentially explaining why heterogeneity becomes more apparent when visceral adiposity is directly assessed.

The use of a composite clinical obesity definition further supports the need to move beyond BMI-only approaches, particularly when evaluating individuals with potentially misclassified adiposity.

### 4.7. Strengths and Limitations

Key strengths include the large sample size, standardized assessments, and simultaneous evaluation of multiple adiposity definitions, allowing comparison of phenotype-specific associations. Limitations include the cross-sectional design (precluding causal inference), self-reported lifestyle measures, and the use of bioimpedance-derived visceral fat estimates, which may not perfectly match imaging-based quantification. Additionally, unmeasured occupational exposures may contribute to residual confounding; long working hours have been associated with obesity risk in dose–response meta-analytic evidence [68], and experimental studies indicate that sleep restriction can alter energy balance and promote visceral obesity [69]. Nonetheless, the coherent gradient of effect sizes across adiposity definitions supports internal consistency.

Although this study was conducted in a relatively homogeneous cohort of young healthcare professionals, the implications of our findings extend beyond this specific population. The observed discrepancies between BMI and alternative adiposity indices, particularly those reflecting central and visceral fat, are consistent with evidence from broader epidemiological studies and highlight a general limitation of BMI as a screening tool. Given the increasing prevalence of obesity and its associated cardiometabolic risks in young adults, reliance on BMI alone may lead to systematic underestimation of clinically relevant adiposity in the general population. From a public health perspective, these findings support the incorporation of complementary anthropometric and body composition measures into routine screening strategies, which may improve early risk detection and enable more targeted preventive interventions at the population level.

Overall, incorporating a clinical definition of obesity based on multiple adiposity indices provides a more robust and clinically meaningful assessment of obesity in young populations.

Recent proposals have emphasized the need to move beyond BMI-based definitions of obesity toward a more comprehensive clinical framework that incorporates adiposity distribution and related health risks. In this context, our findings are aligned with these emerging perspectives, as we demonstrate that BMI alone substantially underestimates clinically relevant adiposity, particularly when central and visceral fat are considered. Although the present study was not designed to apply a formal clinical definition of obesity based on these newer frameworks, the use of multiple adiposity indices provides a complementary approach that captures different dimensions of obesity and supports a more nuanced assessment of cardiometabolic risk.

## 5. Conclusions

BMI-based screening may underestimate clinically relevant central and visceral adiposity in young healthcare professionals. Waist-based and visceral measures appear more sensitive to lifestyle exposures, particularly physical inactivity, and may better capture metabolically adverse phenotypes. Incorporating central/visceral adiposity assessment alongside BMI could improve early risk identification and support targeted prevention strategies in occupational health settings.

## Figures and Tables

**Table 1:** Sociodemographic, Clinical Characteristics, and Health Habits of the Study Population According to Sex and Professional Group.

Variable	Men Physician (*n* = 1862)	Men Nurse (*n* = 612)	Men Control (*n* = 1590)	Women Physician (*n* = 3076)	Women Nurse (*n* = 2374)	Women Control (*n* = 3360)	*p* Sex	*p* Group	*p* Sex × Group
Age (years)	25.9 (1.6)	23.4 (2.3)	26.6 (2.3)	25.9 (1.4)	23.4 (2.3)	26.5 (2.1)	<0.001	<0.001	0.94
Height (cm)	177.0 (7.3)	177.9 (5.3)	176.4 (6.9)	164.7 (6.0)	162.9 (5.8)	163.4 (6.6)	<0.001	<0.001	0.08
Weight (kg)	72.8 (11.3)	76.1 (10.9)	77.9 (11.8)	56.6 (8.0)	59.6 (9.4)	62.1 (13.6)	<0.001	<0.001	0.01
BMI	23.1 (2.9)	24.0 (3.4)	25.0 (3.2)	20.8 (2.5)	22.5 (3.3)	23.2 (4.5)	<0.001	<0.001	0.02
Waist (cm)	79.4 (7.5)	81.4 (9.0)	84.7 (8.0)	68.4 (6.0)	70.0 (7.8)	72.0 (7.2)	<0.001	<0.001	0.01
Hip (cm)	98.0 (7.9)	98.3 (7.2)	97.4 (8.4)	94.1 (8.4)	95.7 (9.1)	94.5 (9.0)	<0.001	0.21	0.15
Systolic BP (mmHg)	120.0 (7.7)	123.2 (4.4)	123.1 (10.8)	111.7 (8.0)	112.2 (6.9)	112.0 (10.7)	<0.001	<0.001	0.03
Diastolic BP (mmHg)	70.4 (7.3)	72.3 (5.7)	71.2 (9.3)	68.7 (6.5)	69.0 (7.0)	69.4 (8.9)	<0.001	<0.001	0.07
Total cholesterol (mg/dL)	162.3 (14.8)	161.3 (22.6)	197.9 (26.5)	164.4 (22.8)	164.6 (25.2)	192.0 (24.8)	<0.001	<0.001	0.12
HDL-cholesterol (mg/dL)	55.0 (7.6)	52.1 (9.1)	53.5 (6.4)	65.0 (13.0)	61.8 (12.2)	55.4 (6.3)	<0.001	<0.001	0.02
LDL-cholesterol (mg/dL)	119.9 (17.5)	123.6 (25.8)	125.5 (25.0)	111.6 (20.0)	115.7 (22.8)	119.6 (26.9)	<0.001	<0.001	0.09
Triglycerides (mg/dL)	62.9 (17.5)	72.5 (28.9)	94.8 (42.5)	61.1 (22.5)	64.7 (22.0)	84.7 (36.3)	<0.001	<0.001	0.04
Glucose (mg/dL)	86.0 (9.5)	86.8 (8.7)	87.1 (10.2)	84.5 (10.0)	84.3 (12.1)	85.1 (10.2)	<0.001	0.03	0.18
AST (U/L)	19.9 (7.0)	19.6 (6.9)	21.8 (7.1)	17.2 (5.4)	17.1 (5.1)	18.8 (6.0)	<0.001	<0.001	0.03
ALT (U/L)	18.0 (6.5)	18.4 (6.4)	19.5 (6.9)	15.1 (7.7)	14.8 (6.9)	16.5 (8.0)	<0.001	<0.001	0.04
GGT (U/L)	17.7 (11.1)	19.3 (5.5)	20.3 (7.8)	15.3 (11.4)	14.8 (7.7)	16.6 (10.1)	<0.001	<0.001	0.05
Smokers (%)	9.1	11.1	25.7	5.7	7.8	27.9	<0.001	<0.001	<0.001
Physical activity (%)	62.2	61.8	58.8	55.2	52.4	53.9	<0.001	0.04	0.21
Mediterranean diet (%)	54.3	52.3	48.8	57.1	56.2	47.6	<0.001	<0.001	0.03

Data are expressed as mean (standard deviation) or percentage (%). Continuous variables were analyzed using two-way ANOVA with sex and professional group as fixed factors, including the sex × group interaction term. Categorical variables were analyzed using log-linear models including sex, professional group, and their interaction. *p*-values for sex, group, and sex × group interaction are shown. When appropriate, post hoc pairwise comparisons between professional groups were performed using Bonferroni correction.

**Table 2:** Anthropometric and Adiposity Indices According to Sex and Professional Group.

Variable	Men Physician	Men Nurse	Men Control	Women Physician	Women Nurse	Women Control	*p* Sex	*p* Group	*p* Sex × Group
BMI	23.1 (2.9)	24.0 (3.4)	25.0 (3.2)	20.8 (2.5)	22.5 (3.3)	23.2 (4.5)	<0.001	<0.001	0.02
WtHR	0.45 (0.04)	0.46 (0.05)	0.48 (0.04)	0.42 (0.03)	0.43 (0.04)	0.44 (0.04)	<0.001	<0.001	0.01
WtHipR	0.81 (0.05)	0.83 (0.06)	0.87 (0.05)	0.73 (0.04)	0.74 (0.05)	0.76 (0.05)	<0.001	<0.001	0.03
CUN-BAE	18.5 (4.2)	20.1 (5.0)	22.8 (4.8)	25.2 (5.1)	27.4 (5.8)	29.1 (6.2)	<0.001	<0.001	0.04
AVI	12.3 (2.1)	13.1 (2.5)	14.8 (2.3)	10.2 (1.8)	10.9 (2.1)	11.7 (2.0)	<0.001	<0.001	0.02
BSI	0.078 (0.006)	0.080 (0.007)	0.083 (0.006)	0.072 (0.005)	0.074 (0.006)	0.076 (0.006)	<0.001	<0.001	0.01
VAI	1.10 (0.45)	1.30 (0.60)	1.80 (0.75)	1.05 (0.50)	1.15 (0.55)	1.60 (0.70)	<0.001	<0.001	0.03
BRI	2.9 (0.6)	3.2 (0.7)	3.8 (0.7)	2.4 (0.5)	2.6 (0.6)	2.9 (0.6)	<0.001	<0.001	0.02
WTGI	3.8 (0.9)	4.2 (1.0)	4.9 (1.1)	3.5 (0.8)	3.7 (0.9)	4.4 (1.0)	<0.001	<0.001	0.04
WWI	10.5 (0.8)	10.8 (0.9)	11.4 (1.0)	9.8 (0.7)	10.1 (0.8)	10.7 (0.9)	<0.001	<0.001	0.03
Body fat (%)	18.2 (5.5)	20.4 (6.1)	24.1 (6.8)	28.5 (6.2)	31.0 (7.0)	34.2 (7.5)	<0.001	<0.001	0.05
Visceral fat (%)	7.5 (2.8)	8.6 (3.1)	10.9 (3.8)	5.2 (2.1)	5.9 (2.4)	7.8 (3.0)	<0.001	<0.001	0.03

BMI: body mass index; WtHR: waist-to-height ratio; WtHipR: waist-to-hip ratio; CUN-BAE: Clínica Universidad de Navarra–Body Adiposity Estimator; AVI: abdominal volume index; BSI: body shape index; VAI: visceral adiposity index; BRI: body roundness index; WTGI: waist triglyceride index; WWI: weight-adjusted waist index. Data are expressed as mean (standard deviation). Continuous variables were analyzed using two-way ANOVA, including sex and professional group as fixed factors, together with the sex × group interaction term. *p*-values for sex, group, and sex × group interaction are shown. When appropriate, post hoc pairwise comparisons were performed using Bonferroni correction.

**Table 3:** Categorical Classification of Obesity According to Different Anthropometric Criteria.

Variable	Men Physician	Men Nurse	Men Control	Women Physician	Women Nurse	Women Control	*p* Sex	*p* Group	*p* Sex × Group
BMI classification (%)							<0.001	<0.001	0.01
Underweight	5.2	0.0	0.0	11.8	6.5	5.8			
Normal weight	69.5	75.2	57.0	80.5	76.5	69.5			
Overweight	24.8	18.3	34.7	7.7	13.6	17.1			
Obesity	0.5	6.5	8.3	0.2	3.4	7.6			
WtHR classification (%)							<0.001	<0.001	0.02
Normal	91.7	79.1	73.2	96.2	93.1	90.4			
High	8.3	20.9	26.8	3.8	6.9	9.6			
WtHipR classification (%)							<0.001	0.02	0.10
Normal	98.0	97.5	96.3	98.7	98.0	97.4			
High	2.0	2.5	3.7	1.3	2.0	2.6			
CUN-BAE classification (%)							<0.001	<0.001	0.03
Normal weight	65.8	64.7	39.5	81.5	63.1	55.8			
Overweight	25.9	19.3	41.0	11.8	22.6	22.5			
Obesity	8.3	16.0	19.5	6.7	14.3	21.7			
Body fat classification (%)							<0.001	<0.001	0.04
Low	18.0	17.6	13.1	46.2	21.9	18.8			
Normal	68.4	67.9	62.3	51.0	68.0	63.8			
High	13.1	13.2	18.5	2.8	8.1	11.8			
Very high	0.5	1.3	6.1	0.0	3.0	5.6			
Visceral fat classification (%)							<0.001	<0.001	0.02
Normal	99.1	98.7	96.5	99.8	99.2	95.6			
High	0.9	1.3	3.5	0.2	0.8	4.4			
Clinical obesity classification (%)							<0.001	<0.001	0.03
Non-clinical obesity	94.5	90.2	85.0	96.8	92.5	87.9			
Clinical obesity	5.5	9.8	15.0	3.2	7.5	12.1			

BMI: body mass index; WtHR: waist-to-height ratio; WtHipR: waist-to-hip ratio; CUN-BAE: Clínica Universidad de Navarra–Body Adiposity Estimator. Data are expressed as percentages. Categorical variables were analyzed using log-linear models including sex, professional group, and obesity definition, together with their interaction terms. *p*-values for sex, group, and sex × group interaction are shown.

**Table 4:** Multivariable-adjusted associations between health behaviors and obesity defined by different adiposity indices.

Variable	BMI Obesity aOR (95% CI)	WtHR aOR (95% CI)	CUN-BAE aOR (95% CI)	Body Fat aOR (95% CI)	Visceral Fat aOR (95% CI)	Clinical Obesity aOR (95% CI)
Age (per year)	1.08 (1.06–1.10)	1.09 (1.07–1.11)	1.10 (1.08–1.12)	1.07 (1.05–1.09)	1.09 (1.07–1.11)	1.10 (1.07–1.13)
Male sex	1.90 (1.70–2.10)	2.10 (1.90–2.30)	2.00 (1.80–2.20)	1.85 (1.65–2.05)	2.20 (2.00–2.40)	2.10 (1.85–2.40)
Nursing residents	1.30 (1.15–1.45)	1.35 (1.20–1.50)	1.40 (1.25–1.55)	1.28 (1.14–1.42)	1.38 (1.24–1.52)	1.45 (1.30–1.62)
Control group	2.80 (2.40–3.20)	3.00 (2.60–3.40)	3.10 (2.70–3.50)	2.75 (2.35–3.15)	3.05 (2.65–3.45)	3.20 (2.70–3.80)
Smoking	1.15 (1.08–1.22)	1.18 (1.10–1.26)	1.20 (1.12–1.28)	1.12 (1.05–1.19)	1.17 (1.10–1.24)	1.20 (1.12–1.28)
Physical inactivity	4.20 (3.70–4.80)	4.50 (3.90–5.10)	4.70 (4.10–5.30)	4.00 (3.50–4.50)	4.60 (4.00–5.20)	4.80 (4.10–5.60)
Mediterranean diet	1.80 (1.60–2.00)	1.90 (1.70–2.10)	2.00 (1.80–2.20)	1.75 (1.55–1.95)	1.95 (1.75–2.15)	2.10 (1.85–2.40)

BMI Body mass index. WtHR Waist to height ratio. BF Body fat. OR Odds ratio. CI: confidence Interval. Values are adjusted odds ratios (aORs) with 95% confidence intervals (95% CIs) from multivariable logistic regression models adjusted for age, sex, professional group, smoking, physical activity, and Mediterranean diet adherence. Reference categories are shown in parentheses.

**Table 5:** Agreement between obesity classifications according to different adiposity indices.

Comparison	Kappa (95% CI)	Interpretation
BMI vs. WtHR	0.45 (0.42–0.48)	Moderate
BMI vs. CUN-BAE	0.55 (0.52–0.58)	Moderate
BMI vs. Body fat	0.60 (0.57–0.63)	Moderate
BMI vs. Visceral fat	0.50 (0.47–0.53)	Moderate
WtHR vs. CUN-BAE	0.58 (0.55–0.61)	Moderate
WtHR vs. Body fat	0.62 (0.59–0.65)	Substantial
WtHR vs. Visceral fat	0.65 (0.62–0.68)	Substantial
CUN-BAE vs. Body fat	0.68 (0.65–0.71)	Substantial
CUN-BAE vs. Visceral fat	0.60 (0.57–0.63)	Moderate
Body fat vs. Visceral fat	0.66 (0.63–0.69)	Substantial
BMI vs. Clinical obesity	0.38 (0.35–0.41)	Fair
WtHR vs. Clinical obesity	0.52 (0.49–0.55)	Moderate
CUN-BAE vs. Clinical obesity	0.58 (0.55–0.61)	Moderate
Body fat vs. Clinical obesity	0.63 (0.60–0.66)	Substantial
Visceral fat vs. Clinical obesity	0.60 (0.57–0.63)	Moderate

Values are Cohen’s kappa coefficients (κ) with 95% confidence intervals. body mass index (BMI), waist-to-height ratio (WtHR), CUN-BAE, body fat percentage, and visceral fat.

**Table 6:** Sex-stratified multivariable-adjusted associations between health behaviors and obesity defined by different adiposity indices.

Variable	Men BMI	Men WtHR	Men CUN-BAE	Men Body Fat	Men Visceral Fat	Men Clinical Obesity	Women BMI	Women WtHR	Women CUN-BAE	Women Body Fat	Women Visceral Fat	Women Clinical Obesity
Age	1.10 (1.06–1.14)	1.11 (1.07–1.15)	1.12 (1.08–1.16)	1.09 (1.05–1.13)	1.11 (1.07–1.15)	1.12 (1.08–1.16)	1.06 (1.03–1.09)	1.07 (1.04–1.10)	1.08 (1.05–1.12)	1.05 (1.02–1.08)	1.07 (1.04–1.10)	1.08 (1.05–1.12)
Nursing	1.40 (1.15–1.70)	1.45 (1.20–1.75)	1.50 (1.25–1.80)	1.38 (1.14–1.65)	1.48 (1.24–1.75)	1.55 (1.30–1.85)	1.25 (1.05–1.48)	1.30 (1.10–1.55)	1.35 (1.15–1.58)	1.22 (1.02–1.45)	1.30 (1.10–1.52)	1.35 (1.15–1.58)
Control	3.00 (2.30–3.90)	3.30 (2.60–4.20)	3.60 (2.80–4.50)	2.90 (2.20–3.70)	3.40 (2.70–4.20)	3.80 (3.00–4.80)	2.20 (1.70–2.80)	2.40 (1.90–3.00)	2.60 (2.00–3.30)	2.10 (1.60–2.70)	2.40 (1.90–3.00)	2.70 (2.10–3.40)
Smoking	1.18 (1.05–1.32)	1.20 (1.08–1.34)	1.25 (1.14–1.38)	1.15 (1.03–1.28)	1.20 (1.08–1.34)	1.25 (1.14–1.38)	1.10 (1.01–1.20)	1.12 (1.03–1.22)	1.15 (1.05–1.26)	1.08 (0.99–1.18)	1.12 (1.03–1.22)	1.15 (1.05–1.26)
Physical inactivity	5.00 (4.00–6.30)	5.20 (4.20–6.60)	5.50 (4.50–6.80)	4.80 (3.80–6.00)	5.30 (4.30–6.60)	5.50 (4.50–6.80)	3.80 (3.00–4.80)	4.00 (3.20–5.00)	4.10 (3.20–5.20)	3.60 (2.90–4.50)	4.00 (3.20–5.00)	4.10 (3.20–5.20)
Mediterranean diet	2.00 (1.70–2.40)	2.10 (1.80–2.50)	2.20 (1.85–2.60)	1.95 (1.65–2.30)	2.10 (1.80–2.50)	2.20 (1.85–2.60)	1.80 (1.55–2.10)	1.85 (1.60–2.15)	1.95 (1.65–2.30)	1.75 (1.50–2.05)	1.85 (1.60–2.15)	1.95 (1.65–2.30)

Values are adjusted odds ratios (aORs) with 95% confidence intervals (95% CIs). Models were adjusted for age, professional group, smoking, physical activity, and Mediterranean diet adherence. body mass index (BMI), waist-to-height ratio (WtHR), CUN-BAE, body fat percentage, and visceral fat.

**Table 7:** Interaction between sex and lifestyle factors in multivariable models of obesity.

Outcome	Sex × Smoking	Sex × Physical Activity	Sex × Mediterranean Diet
BMI obesity	0.08	0.12	0.15
High WtHR	0.04	0.09	0.11
CUN-BAE obesity	0.10	0.07	0.14
Very high body fat %	0.09	0.05	0.13
High visceral fat	0.002	0.001	0.01

Values are *p*-values for interaction terms (sex × exposure) derived from fully adjusted logistic regression models. body mass index (BMI), waist-to-height ratio (WtHR), CUN-BAE, body fat percentage, and visceral fat.

## Data Availability

This manuscript was prepared in accordance with the Strengthening the Reporting of Observational Studies in Epidemiology (STROBE) guidelines for cross-sectional studies. The original contributions presented in this study are included in the article. Further inquiries can be directed to the corresponding author.
